# Radiofrequency Thermoablation of HCC Larger Than 3 cm and Less Than 5 cm Proximal to the Gallbladder without Gallbladder Isolation: A Single Center Experience

**DOI:** 10.1155/2014/896527

**Published:** 2014-08-28

**Authors:** Antonio Orlacchio, Fabrizio Chegai, Costantino Del Giudice, Mariangela Massaccesi, Elisa Costanzo, Elena Di Caprera, Giovanni Simonetti

**Affiliations:** Department of Diagnostic and Molecular Imaging, Radiation Therapy and Interventional Radiology, University Hospital “Policlinico Tor Vergata,” Viale Oxford 81, 00133 Rome, Italy

## Abstract

Radiofrequency ablation (RFA) is an effective minimally invasive treatment for nonsurgical hepatocellular carcinoma (HCC), but ablation of tumors close to the gallbladder could be associated with several complications. We report our experience on the treatment of HCC close to the gallbladder with RFA. Eight RFA procedures were performed in eight patients with HCC larger than 3 cm and less than 5 cm close to the gallbladder. In all cases, a percutaneous approach was used. There were no major complications. Only in two patients a minimal wall thickening of the gallbladder was observed. Contrast enhanced computed tomography carried out after 30 days from the first procedure showed complete necrosis in seven patients (87%). Only one patient had local recurrence at 11 months of followup. Although limited, our experience suggests that, after careful preprocedural planning, in experienced hands and with appropriate technology, percutaneous RFA could be safely performed even for lesions larger than 3 cm located in close adjacency to the gallbladder.

## 1. Introduction

Hepatocellular carcinoma (HCC) is a leading cause of cancer-related death worldwide, and the burden of this devastating cancer is expected to increase further in coming years [1]. Although surgical resection and liver transplant are considered the gold-standard treatment modalities for HCC, their use is limited by the liver function of patients and lack of donors [2]. Nowadays nonsurgical treatments such as radiofrequency thermoablation (RFA) have been widely accepted as effective means of minimally invasive treatment for nonsurgical HCC [3]. Nevertheless peripheral tumors adjacent to extrahepatic organs were also suggested to be unsuitable because of the risk of heat injury, such as intestinal perforation and pleural effusion. Moreover when these techniques are used to treat tumors that are located in proximity of anatomic structures that might be injured by the thermal process, such as gallbladder, complete tumor removal is difficult to achieve without incurring the serious risk of causing necrosis or perforation of the wall of the organ [4]. For the above reason, patients with lesions in the so-called high risk location could be commonly excluded from interventional procedures.

Only a few authors suggested that percutaneous treatment can be safely used to ablate tumors close to the gallbladder with some expedients. For example, a more cautious approach can be that of using multiple ethanol injections to eliminate the residual tumor adjacent to the gallbladder after RFA [5]. Others suggest treating these lesions after the injection of sterilized solution into the gallbladder fossa to space out the tumor from the gallbladder [4]. Recently Jiang et al. reported their experience with RFA without gallbladder isolation assisted by a laparoscopic approach [6]. In this study we reported our experience in 8 patients with HCC smaller than 5 cm located in the so-called high risk site that were treated with percutaneous RFA.

## 2. Materials and Methods

**Figure 1:**
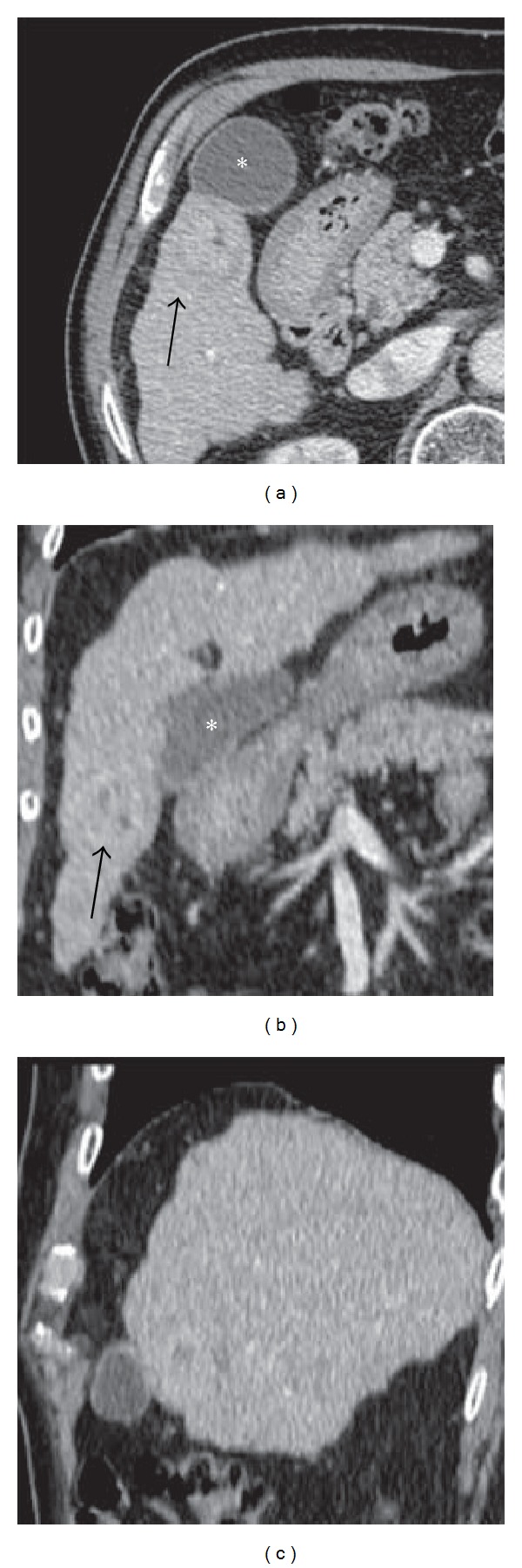
CT Preprocedural axial image (a) and multiplanar reconstructions (b, c) show the relationship between hepatocellular carcinoma (black arrow) and the gallbladder (∗).

From December 2010 to October 2013 we performed percutaneous RFA in eight patients (5 men and 3 women) with liver cirrhosis and with HCC close to the gallbladder (<1 cm in distance). The mean tumor size was 3.2 cm (range: 2.4–4 cm) and tumor edges were near gallbladder wall from 10 to 0 mm. All patients in our study were selected during the followup for liver cirrhosis and the choice of the percutaneous ablative treatment was carried out by the evaluation of a multidisciplinary team of hepatologists, surgeons, and interventional radiologists [7]. The diagnosis of HCC was carried out in accordance with the guidelines of the European Association for the Study of the Liver (EASL) [8] for the diagnosis of HCC in cirrhotic patients. Tumor diameters ranged between 3.0 and 5 cm (Figure 1). The age of patients ranged from 52 to 78 years (mean ± SD: 72.43 ± 5.66). Five patients were in Child-Pugh class A and 3 in class B. The α-fetoprotein levels, registered before the procedure, were on average 561.67 ± 684 ng/mL (range: 25–1870).

Six tumors were located in segment V and two in segment IV. We used RFA and the ablations were performed and monitored according to the recommendations of the equipment manufacturers. Radiofrequency devices used were a 200 W RF generator and an impedance-based multiple-electrode RFA system (Boston Scientific Corporation, Natick, MA, USA). RFA was conducted using an expandable 15 G LeVeen needle, 15 cm long insulated cannula that contained 10 individual hook-shaped electrode arms (Boston Scientific Corporation, Natick, MA, USA). The needles had a maximum diameter of 50 mm when fully deployed.

We performed all liver thermal ablations using an expandable multielectrode system because we think that it is easier to evaluate the position of hooks after their deployment and to predict the volume of thermal effect more accurately with multielectrode system rather than with cool-tip needle. Furthermore the needle position is not influenced by respiratory movements.

The aim of performing radiofrequency ablation in all patients was complete destruction of the lesion with a 5 to 10 mm wide tumor-free margin around all possible aspects of each tumor. The RFA were performed in a single session for all patients. Conscious sedation and local anesthesia were used every time. All ablations were carried out percutaneously under ultrasound and computed tomography guidance. The procedure was performed with conscious sedation and local anesthesia. The routes of electrode insertion were carefully selected according to the feasibility of the needle path and the size and the shape of the lesion.

Needle introduction was performed, depending on the location of the nodule to be treated, in 4 patients through subcostal approach and in 4 patients through intercostal approach. To better conform the shape of the ablated volume to the tumor while reducing the risk of gallbladder perforation by the hook-shaped electrode arms of the device, we decided to insert the needle through the liver surface towards the gallbladder, perpendicularly to the gallbladder right wall.

Before the deploying of the hooks, the distance between the tip of the needle and the gallbladder wall was accurately checked and kept away from gallbladder wall to avoid direct or thermal injuries.

The efficacy of treatment was assessed by dynamic triphasic CT, 30 days after the procedure. The treatment response was evaluated according to mRECIST criteria [9]. Complete response (CR) was considered the disappearance of any intratumoral arterial enhancement at CT evaluation; partial response (PR) was at least a 30% decrease of the longest diameter of the viable lesion evaluate as contrast enhancement in the arterial phase respect to the preprocedural CT result. Three-phase dynamic CT scans in the 3rd, 6th, and 12th months followup were performed for all patients.

Intraprocedural and postprocedural complications were evaluated according to the classification of the International Society of Radiology (SIR, Society of Interventional Radiology) [10] distinguishing between major (events that lead to substantial morbidity and disability, an increase of care, hospitalization, or a longer hospital stay) and minor complications. The presence of the “postablation syndrome,” defined as fever, nausea, vomiting, and localized pain in the abdomen and referred to the shoulder, was investigated during the first 48 h after treatment.

Moreover the CT scans obtained before and immediately after ablation and subsequently were reviewed by one experienced radiologist for the presence of gallbladder wall thickening of more than 2 mm, abnormal gallbladder wall enhancement, and pericholecystic fluid.

## 3. Results

In our experience there were no cases of treatment-related deaths. In addition, there were no major complications such as cholecystitis or gallbladder perforation. A minimal wall thickening of the gallbladder (2.0-3.0 mm, *n* = 9) was seen as focal wall enhancement adjacent to the RF ablation zone in 2 patients. All two cases of gallbladder wall thickening after ablation were associated with symptoms.

Postablative syndrome was observed in 6 patients, manifested by fever (four patients), chills (two patients), and localized pain in the abdomen (three patients). All patients reported various degrees of malaise in the days following the procedure such as asthenia, weakness that appeared three to five days after the procedure and lasted on average four days.

**Figure 2:**
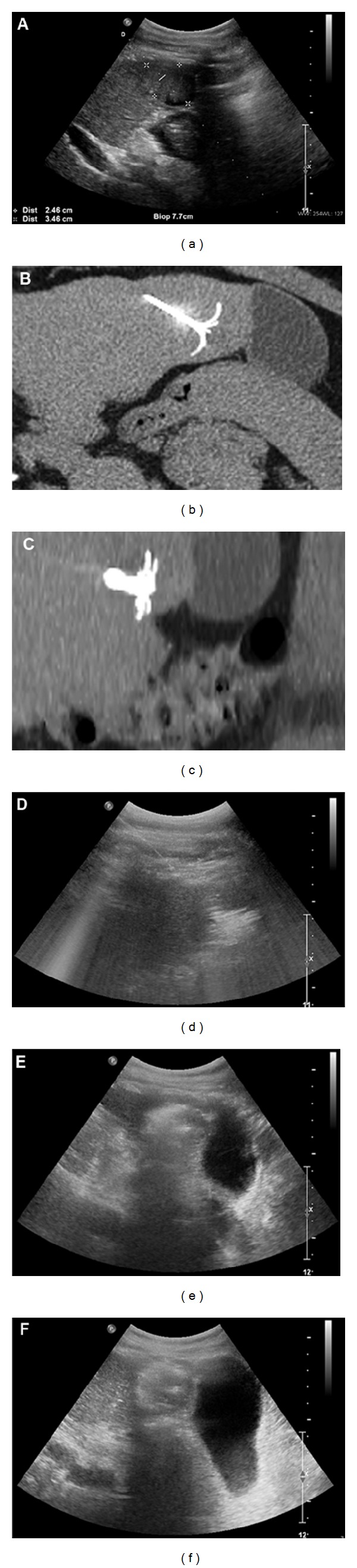
Radiofrequency ablation of the hepatocellular carcinoma performed under US and CT control with an expandable needle. (a) US view shows the hepatocellular carcinoma and the planning of the needle insertion in the liver lesion. CT axial (b) and reformatted (c) images depict the position of the needle inside the lesion. (d) US control during radiofrequency shows the hyperechoic zone that covers the entire liver lesion. US controls at the end of the first roll-off (e) and at the end of the procedure (f) do not depict any gallbladder anomalies.

No procedure has been interrupted and technical success was obtained in 100% of patients. In all patients, during RFA, a slowly enlarging and coalescing hyperechoic zone appeared around the distal tip of the needle, resulting from vaporization of fluid and formation of microbubbles of gas (Figure 2). At the end of the treatment, an irregular and poorly defined echogenic zone occupied the whole treated area. At the end of the procedures, after the automatic cool-down of the RF system, the generator was reactivated during the needle retraction to prevent the tumor dissemination and to permit the coagulation of the needle channel.

CT control after 30 days from the first procedure showed complete necrosis (no detectable remaining tumor on CT) in seven patients (87%). In the remaining patient, the lesion was treated with chemoembolization.

The follow-up times ranged from 12 to 18 months (mean: 15 months) in the patients with completed ablations.

**Figure 3:**
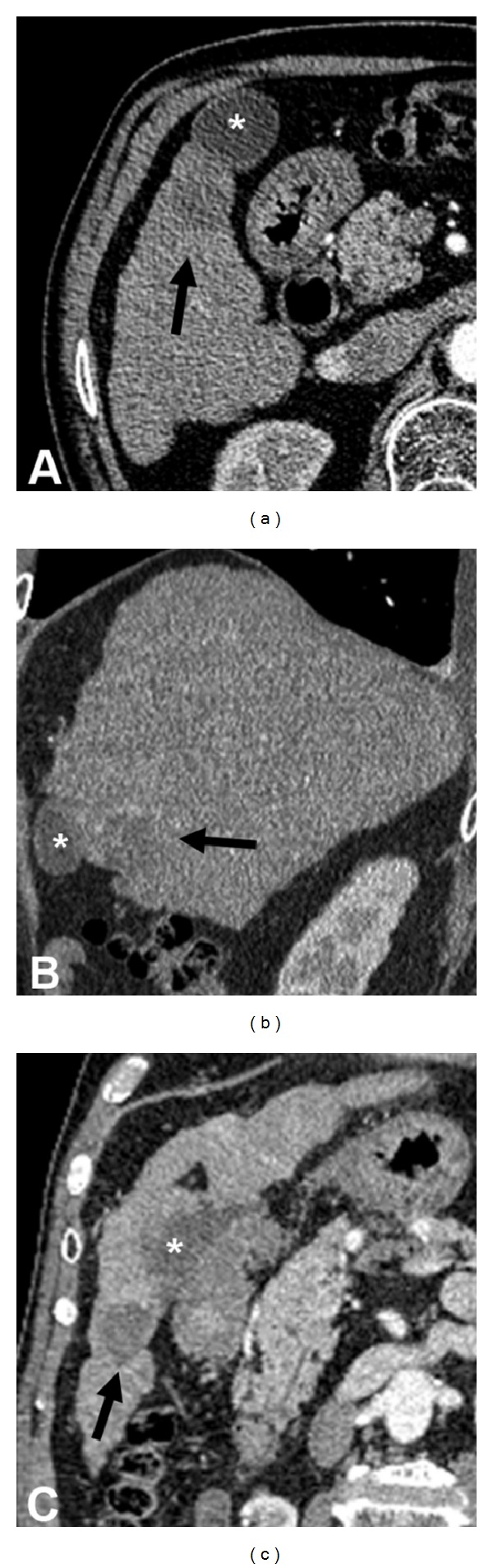
Axial CT image (a) and CT multiplanar reconstructions (b, c), performed after six months, show the absence of contrast medium enhancement of the ablated liver lesion (black arrow) without gallbladder (∗) damage.

One patient had local recurrence at 11 months. The remaining six patients showed no local recurrence at the end of the follow-up period (Figure 3). None of the patients showed evidence of gallbladder disease.

## 4. Discussion

RFA is a minimally invasive method used to destroy tumors within solid organs and it has been used for primary liver tumors and for hepatic metastases [11, 12]. Nevertheless, RFA of tumors adjacent to the gallbladder is often accompanied by high risk of gallbladder perforation, acute cholecystitis, or postoperative bleeding [13, 14]. Furthermore, combination therapy or a secondary round of RFA treatment is often needed to achieve complete tumor eradication [15]. To separate the liver tissue from the gallbladder and to prevent gallbladder injuries during thermal ablation, Cirocchi and colleagues suggested the injection of 40–80 mL of aseptic solution into the gallbladder fossa and cystic plate just before the RFA or the use of a “lift-expand” technique to confirm that the needle tip does not stick into the gallbladder wall [11]. Recently Jiang and coworkers suggested that tumor next to the gallbladder can be safely treated with RFA with laparoscopic guidance without the need of removing or isolating the gallbladder [6].

Although these methods of gallbladder isolation and laparoscopic RFA appear to be safe, they may increase the duration time of the procedure, may have an influence on health care costs of the examination, and may involve additional risks for patients [11].

In this report we described eight cases of HCC adjacent to the gallbladder that were successfully treated with percutaneous RFA without gallbladder isolation. Previously Chopra and colleagues [15] conducted a study to assess the feasibility and safety of RFA of hepatic tumors (primitive or metastatic) with a maximum diameter of less than 3 cm, adjacent to the gallbladder. The authors used RFA with an expandable electrode to treat 8 patients (2 patients with HCC and 6 patients with liver metastases). As assessed by CE-CT scan after one month from the procedure, a complete ablation was achieved in all patients except one (87%). Except for gallbladder wall thickening, no complications were reported. The authors concluded that radiofrequency ablation of hepatic tumors adjacent to the gallbladder is feasible and potentially safe for lesions with a maximum diameter less than 3 cm. Our results not only confirm those by Chopra and coworkers but also suggest the feasibility of RFA even in HCC adjacent to the gallbladder larger than 3 cm and less than 5 cm.

To this purpose, a proper knowledge of the available resources and careful preprocedural planning were mandatory.

We decided to use RFA instead of other percutaneous procedures (e.g., percutaneous laser ablation and microwave ablation) because of the opportunity to monitor in real time the necrosis of the tissue, which makes this technique particularly suitable for the treatment of lesions located in the close proximity of critical structures. Indeed, while during percutaneous laser ablation or microwave ablation there are no reliable methods to monitor the evolving necrosis of the hepatic tissue, thus resulting in a wide variability of the amount of liver tissue ablated with different protocols [16], during RFA it is possible to exactly detect the occurrence of the necrosis by monitoring the electrical impedance (Ohm) of the hepatic tissue during the delivery of RF energy. Tissue desiccation causes an increase of the impedance within the ablated volume that prohibits the passage of electrical current and leads the power output from the generator to fall to zero (i.e., roll-off) [17]. In this way it is possible to reproduce a constant volume of necrosis.

The correct selection of the appropriate device to perform the tumor ablation was also a critical issue.

Teratani and colleagues [18] treated 207 patients with hepatocellular carcinoma (HCC) in presumably high risk locations, using a single probe cool-tip electrode, declaring that the use of multiprobe system with extension of the hooks could not be precisely controllable when applied to nodules adjacent to large vessels or extrahepatic organs. Nevertheless we choose an expandable multiple-electrode system and we decided to insert the needle at a direction perpendicular to the gallbladder wall in order to prevent, during the opening of the hooks, the organ perforation. In this way we were sure that the hooks would be positioned in the safety position, according to three spatial dimensions.

## 5. Conclusions

Although limited, our experience suggests that percutaneous RFA is feasible even for lesions larger than 3 cm located in close adjacency to the gallbladder. Careful preprocedural planning with a proper knowledge of the available technology is mandatory. Obviously, the promising result in these three cases needs to be confirmed in larger series of patients with lesions next to critical structures.
